# Online information-seeking behavior of Iranian web users on Google about Henoch–Schönlein purpura (HSP): an infodemiology study

**DOI:** 10.1186/s12913-023-10357-2

**Published:** 2023-12-11

**Authors:** Vadood Javadi, Sharareh Kamfar, Vahide Zeinali, Khosro Rahmani, Foroughossadat Hosseini Moghaddamemami

**Affiliations:** 1 Pediatric Pathology Research Center, Research Institute for Children’s Health, Shahid Beheshti University of Medical Sciences, Tehran, Iran; 2 Pediatric Congenital Hematologic Disorders Research Center, Research Institute for Children’s Health, Shahid Beheshti University of Medical Sciences, Tehran, Iran; 3 Department of pediatric rheumatology, Shahid Beheshti University of Medical Sciences, Mofid children’s Hospital, Tehran, Iran; 4 Department of Pediatric Rheumatology, School of Medicine, Guilan University of Medical Sciences, Rasht, Iran

**Keywords:** Henoch–Schönlein purpura, Information-seeking behavior, Health information, Infodemiology, Web users, Google

## Abstract

**Backgrounds:**

: Previous studies have indicated that users’ health information-seeking behavior can serve as a reflection of current health issues within a community. This study aimed to investigate the online information-seeking behavior of Iranian web users on Google about Henoch–Schönlein purpura (HSP).

**Methods:**

Google Trends (GTr) was utilized to collect big data from the internet searches conducted by Iranian web users. A focus group discussion was employed to identify users’ selected keywords when searching for HSP. Additionally, keywords related to the disease’s symptoms were selected based on recent clinical studies. All keywords were queried in GTr from January 1, 2012 to October 30, 2022. The outputs were saved in an Excel format and analyzed using SPSS.

**Results:**

The highest and lowest search rates of HSP were recorded in winter and summer, respectively. There was a significant positive correlation between HSP search rates and the terms “joint pain” (P = 0.007), “vomiting” (P = 0.032), “hands and feet swelling” (P = 0.041) and “seizure” (P < 0.001).

**Conclusion:**

The findings were in accordance with clinical facts about HSP, such as its seasonal pattern and accompanying symptoms. It appears that the information-seeking behavior of Iranian users regarding HSP can provide valuable insights into the outbreak of this disease in Iran.

**Supplementary Information:**

The online version contains supplementary material available at 10.1186/s12913-023-10357-2.

## Introduction

The use of the Internet is increasing globally. Approximately 4.4 billion people around the world (60% of the world’s total population) use the Internet and spend almost 7 h online each day [1]. All human activities, such as communication, education, and shopping, have been affected by the Internet [2]. Additionally, it is one of the most crucial information sources that people use to find information in particular health information. The accuracy, comprehensiveness, and readability of online health information have been challenged by many studies [3–5]. However, many people first consult the Internet before seeking a professional diagnosis or use it as a source of information alongside other providers such as physicians and health professionals [6].

The increasing tendency of people to search the Internet for health information, accompanied by information technology capabilities such as big data and data mining tools, has enabled researchers to track health information trends and monitor the search behavior of web users about specific diseases and health problems [7]. The field of research that includes studying the distribution and determinants of information in an electronic medium, specifically the Internet, has been known as infodemiology [8]. Infodemiology, formed from the fusion of “information” and “epidemiology,“ represents an interdisciplinary domain that incorporates elements from epidemiology, information science, and computer science. Epidemiology is a field of medical science focused on the determinants, occurrence, and distribution of health and disease in a defined population [9]. Infodemiology aligns with epidemiology in specific objectives, such as tracking public health patterns and advocating for health interventions [10].

The first infodemiology study was published in 2006 by Eysenbach that showed a correlation between influenza-related searches on Google and influenza cases occurring in the following week in Canada [11]. After that, Ginsberg and colleagues reported the same findings of the prediction of influenza by monitoring search queries on Google in the United States [12]. Pelat and colleagues also used the same method to predict the prevalence of three infectious diseases: influenza, diarrhea, and smallpox. Their research showed that the search behavior of Google users has a high aptitude to predict the prevalence of diseases [13].

Today, there are a growing number of studies that investigate the relationship between Internet search behavior and the incidence or prevalence of various infectious diseases such as ebola [14], syphilis [15], dengue fever [16], HIV [17], and COVID-19 [18]. Some studies have used infodemiology methods in estimating the prevalence of non-infectious diseases. For example, Sciascia and colleagues used infodemiology to investigate the prevalence of antiphospholipid syndrome as a rare disease [19]. Some studies used infodemiology to monitor and predict the incidence and prevalence of cancers, as well [20, 21]. It must be noted that predicting the disease outbreak is not the only application of infodemiology. The infodemiology data can also be used to evaluate health information availability, public health-relevant publications, and the effectiveness of health marketing campaigns in different communities [8].

Henoch–Schönlein purpura (HSP), also known as IgA vasculitis, is the most common acute vasculitis characterized by non-thrombocytopenic purpura, arthritis or arthralgia, abdominal pain, and renal involvement [22]. HSP occurs throughout the year, but many studies have noted a tangible increase in cases from fall through spring and a decrease during the summer [23, 24]. Most studies on the incidence of HSP have been performed in European countries [25, 26], and there is little information about the incidence of this disease in other countries of the world. For example, there is no reliable information about the prevalence and incidence of HSP in Iran. Determining the prevalence and incidence of HSP in Iran requires extensive and long-term epidemiological studies. Conducting such studies needs financial support and the participation of various medical organizations throughout the country. According to the findings of previous studies about the ability of infodemiology to predict the prevalence of various diseases [11–18, 20, 21], this study aimed to investigate the online information-seeking behavior of Iranian users in Google about HSP. Due to the cost and time-consuming nature of epidemiological studies, the present study can provide a less expensive, real-time, and estimated view of the outbreak of HSP in different provinces of Iran. Also, since the incidence of HSP follows a seasonal pattern, the results of the present study can be used to evaluate the accuracy of infodemiology data in predicting the incidence of diseases.

## Methods

### Keywords selection

In this cross-sectional study, a focus group discussion was used to identify users’ selected keywords during a search for HSP. A telegram group consisting of patients with HSP and their caregivers was formed. They were asked about their history of online searching the information about HSP, and the keywords they used for the search. Based on the collected data, three keywords had a high frequency: “Henoch–Schönlein purpura,” “Henoch,” and “Bimari Henoch.” The keywords were in Persian. The keywords related to the symptoms of the disease were selected based on recent clinical studies [27, 28] and translated to Persian by rheumatologists. The symptoms’ keywords were “Joint pain,” “Joint swelling,” “Hands and feet swelling” for joint involvements; “Abdominal pain,” “Nausea,” “Vomiting,” “Bloody vomiting,” “Stool discoloration,” “Black stools,” “Bloody stools” for gastrointestinal involvements; “Skin rash,” “Red spots,” “Hives” for purpura; “Bloody urine” for renal involvement; “Seizure” for nervous involvement; and “Inflammation of the testicles.”

### Search strategy

Google Trends (GTr) was used for gathering the big data from the internet searches of Iranian web users. It is an open online tool (https://trends.google.com/trends/) that allows tracking of millions of searches on the Google search engine. GTr algorithms, by normalizing data, display search rates based on a scale from zero (if the search rate is less than 1% of the maximum related searches) to 100 (highest popularity). This platform can display the frequency of searches by geographical regions in weekly, monthly, and annual intervals [29]. The keywords were queried in GTr on November 15, 2022. The terms were not used in combination and were queried with quotation marks. In this study for searching Google trends, the geographic area of the search was limited to Iran. Also, to boost the relevancy of results, the category of the search was set to “Health” and “Web search.“ The time duration of all searches was limited from January 1, 2012, to October 30, 2022.

### Statistical analysis

The outputs were saved in an Excel format and analyzed using SPSS (Version 23). A one-way repeated measures ANOVA was conducted to evaluate the effect of time on HSP-related search rates. Additionally, one-way ANOVA was used to assess the seasonal pattern of Iranians’ HSP-related searches. Pearson’s correlation coefficient was employed to examine the correlation between the search rates of main keywords and symptom-related keywords. Furthermore, Spearman’s correlation coefficient was used to assess the correlation between keyword search rates and the populations of various provinces. In all statistical analyses, the significance level was set at p < 0.05.

## Results

**Fig. 1:**
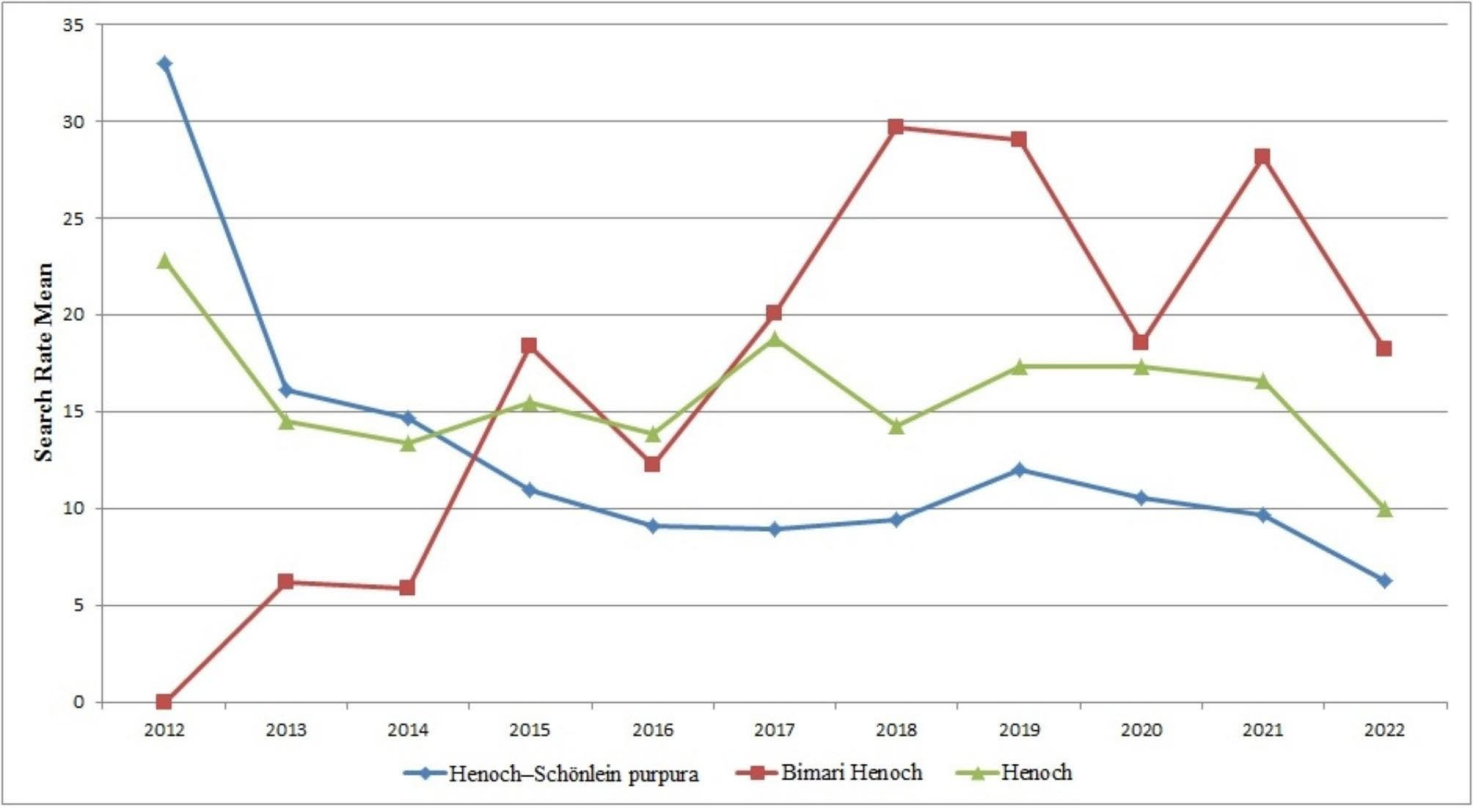
The search rate of Iranian users for “Henoch”, “Bimari Henoch”, and “Henoch–Schönlein purpura” on Google

The search rate of “Henoch,” “Bimari Henoch,” and “Henoch–Schönlein purpura” had some fluctuations from 2012 to 2022. There was no constant increase or decrease in search rate on any keyword. The highest search rate for “Henoch” and “Henoch–Schönlein purpura” occurred in 2012. The search rate of “Henoch–Schönlein purpura” had a sensible decrease from 2012 to 2016. It continued its downward trend after a slight growth in 2018 and 2019. The search rate of “Henoch” followed a similar pattern with fluctuations in 2015, 2017, and 2019. The searches for “Bimari Henoch” began in 2013 and peaked after some fluctuations in 2018. Despite numerous fluctuations, the search rate of “Bimari Henoch” was higher than the other keywords from 2017 to 2022 (Fig. 1).

**Fig. 2:**
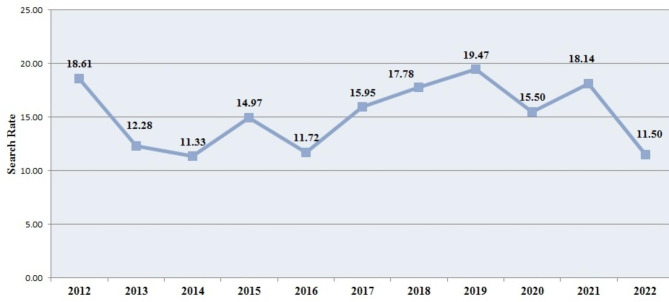
The total search rate of Iranian users for HSP related information on Google

The total search rate of HSP on Google for each year was determined by calculating the mean of three keywords’ search volumes (Fig. 2). According to the results of the analysis, time did not have a significant effect on the overall trend of searches performed by Iranian users about HSP; *F (10,119) = 1.306 P-value = 0.23.* The highest and lowest search volumes of HSP on Google were recorded in 2019 and 2014, respectively.

**Fig. 3:**
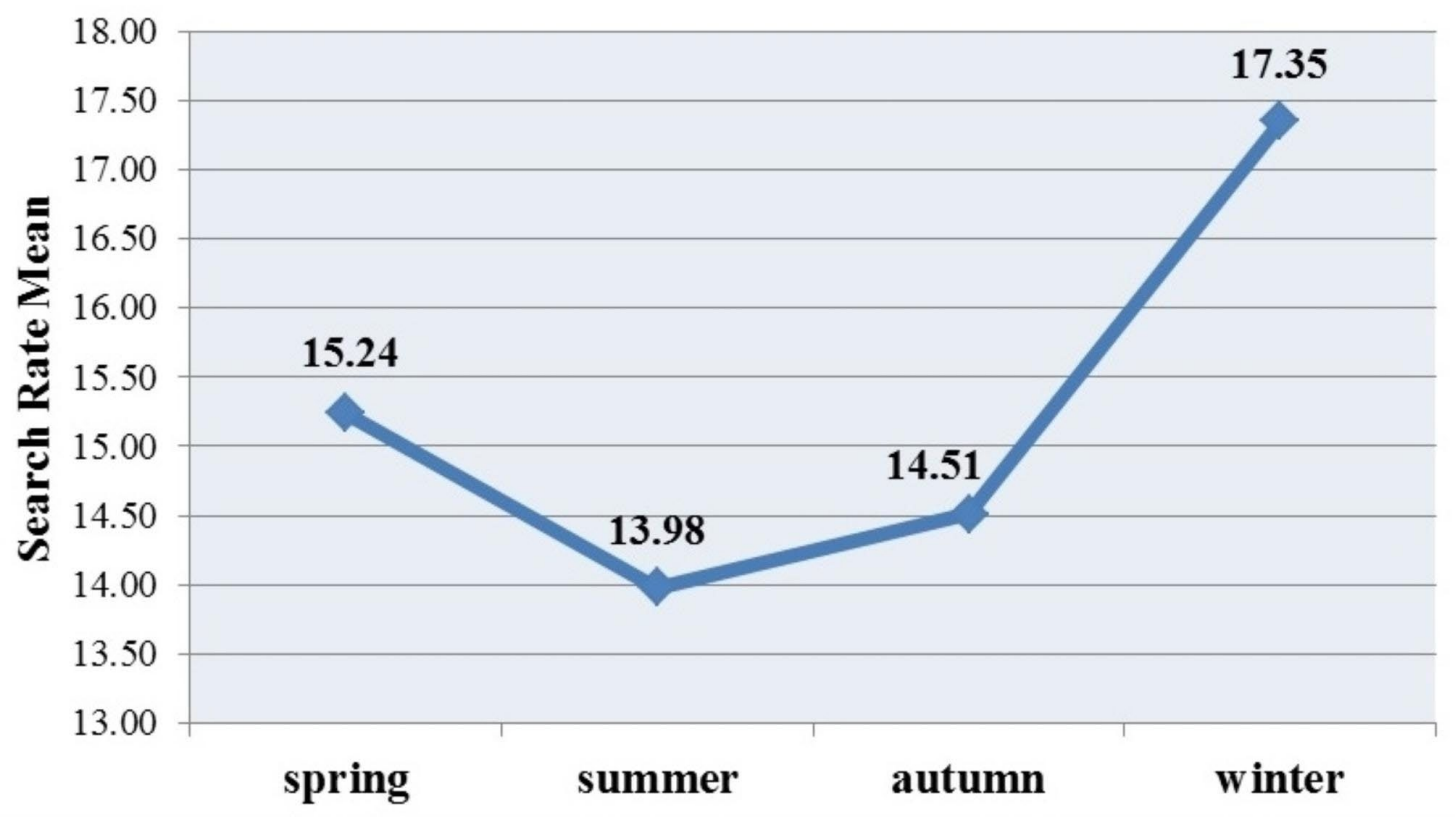
Total search rate of HSP in different seasons

There was not a significant relationship between the search rate of the three main keywords and the seasons (P > 0.05). Also, there was no significant relationship between the total search rate of HSP and the seasons (Table 1). However, the highest search rate of HSP-related information was recorded in winter, spring, and autumn, respectively (Fig. 3).

**Table 1:** The relationship between search rate of HSP keywords and seasons

	Search rate mean ± standard deviation				P-value
	Spring	Summer	Autumn	Winter	
Henoch–Schönlein purpura	15.87 ± 17.54	10.48 ± 7.83	12.00 ± 6.60	13.21 ± 9.39	0.25
Bimari Henoch	17.18 ± 23.22	13.90 ± 15.36	18.53 ± 18.96	18.21 ± 22.59	0.78
Henoch	12.66 ± 11.43	17.54 ± 17.11	13.00 ± 10.59	20.62 ± 21.53	0.13
Total HSP related searches	15.24 ± 9.26	13.98 ± 9.00	14.51 ± 8.70	17.35 ± 10.41	0.49

There was a positive and significant correlation between the total rate of HSP related searches and the search rate of “joint pain,” “vomiting,” “hands and feet swelling,” and “seizure” (P < 0.05) (Table 2).

**Table 2:** Correlation between the search volume of HSP main keywords and symptoms

Symptoms	Henoch–Schönlein purpura		Bimari Henoch		Henoch		Total HSP related searches	
	r	Sig	r	Sig	r	Sig	r	Sig
Joint pain	0.315	0.000	0.206	0.019	− 0.072	0.418	**0.234**	0.007
Joint swelling	− 0.006	0.942	0.007	0.939	− 0.002	0.982	0.001	0.991
Abdominal pain	− 0.362	0.000	0.419	0.000	− 0.033	0.713	0.136	0.122
Skin rash	− 0.321	0.000	0.162	0.065	− 0.090	0.311	− 0.064	0.471
Nausea	− 0.370	0.000	0.426	0.000	− 0.010	0.907	0.151	0.087
Vomiting	− 0.305	0.000	0.442	0.000	− 0.011	0.900	**0.188**	0.032
Bloody urine	− 0.297	0.001	0.415	0.000	− 0.031	0.724	0.161	0.067
Bloody vomiting	− 0.135	0.126	0.192	0.029	− 0.055	0.536	0.052	0.556
Hands and feet swelling	0.001	0.993	0.198	0.024	0.065	0.460	**0.180**	0.041
Stool discoloration	− 0.312	0.000	0.146	0.097	0.026	0.766	− 0.005	0.951
Black stools	− 0.333	0.000	0.333	0.000	− 0.018	0.838	0.095	0.282
Bloody stools	− 0.313	0.000	0.293	0.001	− 0.112	0.206	0.021	0.813
Red spots	− 0.193	0.028	0.084	0.345	0.028	0.753	− 0.002	0.983
Seizure	− 0.073	0.412	0.397	0.000	0.103	0.245	**0.314**	0.000
Inflammation of the testicles	0.073	0.410	− 0.210	0.817	0.130	0.139	0.089	0.315
Hives	− 0.342	0.000	0.377	0.000	0.031	0.725	0.151	0.086

**Fig. 4:**
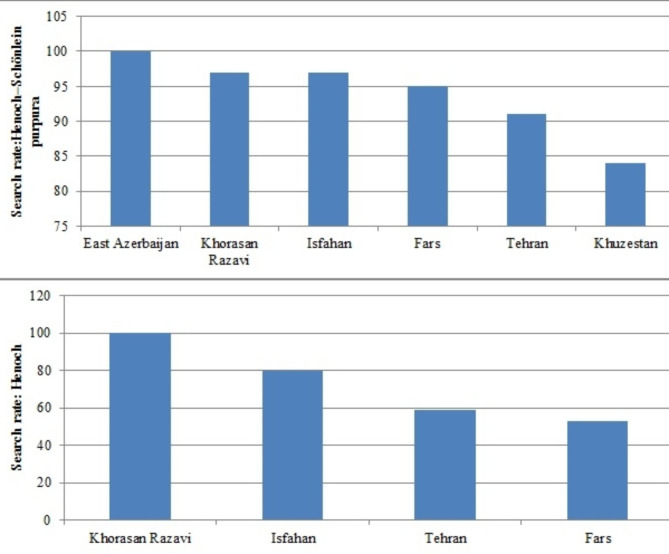
The search rate of “Henoch–Schönlein purpura” and “Henoch” in provinces of Iran

Figure 4 shows the distribution of keywords’ search rates by the province where the users live. It includes the search rate of “Henoch–Schönlein purpura” and “Henoch.” GTr did not have a significant output for “Bimari Henoch.” This is because GTr processes the keywords that have a certain amount of search volume index. According to GTr outputs, “Henoch–Schönlein purpura” had a high rate of search in East Azarbaijan, Khorasan Razavi, Isfahan, Fars, Tehran, and Khuzestan, respectively. Also, the search rate of “Henoch” was considerable in Khorasan Razavi, Isfahan, Tehran, and Fars. The search rate of the keywords in other provinces was not reported by GTr. It means that the search volume had been lower than the index level. The national census report of Iran was used to examine the correlation between the population of the provinces and the keywords’ search volume. There was no significant correlation between the population of provinces and the search rate of “Henoch–Schönlein purpura” (r=-0.241 P = 0.646) and “Henoch” (r=-0.288 P = 0.712) (Supplementary Table 1).

## Discussion

Infodemiology, employing innovative methods, plays a pivotal role in health informatics research across various medical fields [30]. Surprisingly, there appears to be a notable absence of studies employing the infodemiology approach within the field of rheumatology in contrast to other medical disciplines. Our investigation found only three relevant studies in rheumatology: One focused on seasonal trends in systemic lupus erythematosus (SLE) through infodemiology [31], another examined the impact of specific events on SLE-related searches [32], and the third used infodemiology to determine terms associated with rheumatology and arthritis in North America [33]. This study applied a similar approach to determine if GTr’s data regarding HSP would show patterns that could be comparable to disease epidemiology.

There is no integrated system for recording the information of the patients with rheumatic disease in Iran. Additionally, a comprehensive epidemiological study has yet to be conducted to determine the prevalence and incidence of HSP in Iran. Therefore, it is not possible to compare the findings of this infodemiology study with epidemiological facts. However, the findings are consistent with some clinical facts about HSP disease. For instance, most clinical studies have reported a seasonal variation in HSP onset, with most patients presenting from fall through spring and a paucity of cases during the summer months [34, 35]. The findings of this study confirm the seasonal skewing, although these changes were not statistically significant. According to the results, the highest volume of searches was performed in winter, which is the most common season for HSP onset [36]. Also, the lowest volume of the search occurred in the summer, similar to the drop in the number of HSP cases during the summer months in clinical studies [26, 37].

The findings were in line with other studies suggesting that GTR’s data can mirror the epidemiological facts about the seasonability of diseases. For instance, Radin and colleagues investigated the seasonability of SLE by infodemiology. They observed a seasonality trend for Google relative search volumes for lupus-related terms with peaks in spring and winter in both hemispheres. These findings were consistent with the results of local epidemiological studies about the prevalence of SLE [31]. Another study using GTr’s data suggested a seasonality of restless symptomatology with a peak in the summer months in both hemispheres. Their findings were in accordance with clinical observations [38]. Additionally, Platek et al. demonstrated seasonal trends in hypertension prevalence in Poland, which were significantly correlated with the search phrase “Hypertension” on Google. Both hypertension and searches for it were more likely to occur during the winter months [39].

According to our findings, there was a positive and significant correlation between the search rate of “joint pain,” “hands and feet swelling,” “vomiting,” and “HSP.” Joint and gastrointestinal involvements are common among patients with HSP [40]. Additionally, many patients or their caregivers search for the symptoms online before consulting a physician [6]. Therefore, the observed correlation was expected. Wang et al. reported a similar correlation between the search volume of “Allergic Rhinitis” and its triggers. They indicated that there was a positive correlation between the search for “Pollen Allergy,” “Mites,” “Dust Mite,” and “Allergic Rhinitis” [41].

Nervous system involvement is less common than other symptoms among patients with HSP [42]. However, there was a positive and significant correlation between the search rate of “seizure” and “HSP.” The findings showed that the search rate of “HSP” was higher in cold months in Iran. On the other hand, the high prevalence of infectious diseases in the winter, potentially causing fever and seizures in patients [43], could be contributing to the increased Google searches for “seizure.” Therefore, the strong and significant correlation may be due to the similar seasonal patterns of these two disorders.

According to the studies, skin rash is the first symptom of HSP that mainly concentrates on the lower extremities and buttocks [35]. However, there was no significant correlation between its search rate and the search rate of “HSP.” It is worth mentioning that skin rash is a common symptom of many diseases. It can occur from a variety of factors, including infections, heat, allergens, immune system disorders, and medications [44]. Therefore, the lack of significant correlation is not strange and irrational.

We also found that the search rates of HSP-related keywords were more than 40 in six provinces in Iran, while the search rates in other provinces remained lower than the index level. (47)(48)All six provinces are among the most populous provinces of Iran. However, no statistically significant relationship was found between the population of these provinces and the search rate for “HSP.“ The search rates were probably influenced by the disease’s prevalence rather than the population of the provinces. These findings can be helpful in providing health policies for the management of HSP in Iran. Furthermore, they can prove invaluable for healthcare practitioners, particularly pediatricians, in these provinces, aiding in the monitoring of HSP prevalence and facilitating HSP health education efforts.

Our findings also indicated that there was no steady decline or increase in the volume of searches about HSP in Iran. If the search rate of the disease reflects its approximate prevalence [11–13], it can be concluded that the HSP outbreak in Iran fluctuated between the years 2012 and 2022. Also, the volume of searches peaked in 2012, 2019, and 2021, with an average search rate below 20 during these peaks. The observed changes were not sudden and did not significantly deviate from the usual search trend. To the best of our knowledge, the peaks did not coincide with special scientific or social events related to HSP. It is essential to note that sudden and significant increases in search volume may be related to specific events or news headlines. Sciascia and Radin’s longitudinal study provided instances where events, such as the Food and Drug Administration’s approval of Belimumab for SLE treatment in March 2011 or the public disclosure of Lady Gaga and Selena Gomez’s SLE diagnoses in June 2010 and October 2015, respectively, led to a sudden escalation in search queries related to SLE on GTr and Wikipedia [32]. Hence, the interpretation of infodemiology outcomes aimed at assessing disease prevalence should adopt a comprehensive perspective, considering various factors that can influence individuals’ online information-seeking behaviors.

This study had some limitations. Because of the nature of GTr, there was no access to demographic data (such as age and sex) of users who conducted the searches. Another limitation was related to the variation of access to the Internet among Iranian users and their information literacy levels. These confounding variables may affect the results of the study. Because of the limitations, while interpreting the data, the cause and effect relationship was not emphasized.

## Conclusion

In summary, our study revealed that the search rate of Iranian web users about HSP fluctuated between 2012 and 2020. However, it followed the seasonal pattern of HSP incidence. Additionally, the search rates of some joint and gastrointestinal symptoms such as ‘joint pain,” “hands and feet swelling,” and “vomiting” had a positive correlation with the search rate of HSP. In the future, conducting epidemiological studies on the prevalence and incidence of HSP in Iran will allow us to identify which keywords can predict upcoming HSP incidences. This study demonstrated that the online information-seeking behavior of Iranian users about HSP on Google was in harmony with clinical data. Consequently, GTr data can be as useful and effective in studies on the outbreak of HSP as in infectious diseases.

### Electronic supplementary material

Below is the link to the electronic supplementary material.

Supplementary Material 1

## Data Availability

The study was based on Google Trends’ data (https://trends.google.com/trends/). The extracted data that support the findings of this study are available at https://www.researchgate.net/publication/372788957_HSP_infodemiology_2012-2022.
