# Supplementary material for: Online information-seeking behavior of Iranian web users on Google about Henoch–Schönlein purpura (HSP): an infodemiology study
Source: BMC Health Serv Res. 2023 Dec 11;23:1389. doi: 10.1186/s12913-023-10357-2 (PMC10714479; doi:10.1186/s12913-023-10357-2)
Supplement: Supplementary file 1 — Supplementary Material 1 [file 12913_2023_10357_MOESM1_ESM.docx]

Supplementary table 1.Correlation between the search volumes of “Henoch–Schönlein purpura,” “Henoch,” and population of provinces

| Provinces | Population | Henoch–Schönlein purpura | | | Henoch | | |
| --- | --- | --- | --- | --- | --- | --- | --- |
|  |  | Search rate | r | Sig | Search rate | r | Sig |
| East Azarbaijan | 3909652 | 100 | -0.241 | 0.646 | - | -0.288 | 0.712 |
| Isfahan | 3265219 | 97 |  |  | 80 |  |  |
| Khorasan Razavi | 6434501 | 97 |  |  | 100 |  |  |
| Fars | 4851274 | 95 |  |  | 53 |  |  |
| Tehran | 13267637 | 91 |  |  | 59 |  |  |
| Khuzestan | 47110509 | 84 |  |  | - |  |  |
